# Phylogenetically-Informed Priorities for Amphibian Conservation

**DOI:** 10.1371/journal.pone.0043912

**Published:** 2012-08-30

**Authors:** Nick J. B. Isaac, David W. Redding, Helen M. Meredith, Kamran Safi

**Affiliations:** 1 Natural Environment Research Council Centre for Ecology and Hydrology, Maclean Building, Wallingford, Oxfordshire, United Kingdom; 2 University College London, London, United Kingdom; 3 Durrell Institute of Conservation and Ecology and Institute of Zoology, Zoological Society of London, London, United Kingdom; 4 Max Planck Institute for Ornithology, Department for Migration and Immuno-ecology, Radolfzell, Germany; 5 Department of Biology, University of Konstanz, Konstanz, Germany; Imperial College Faculty of Medicine, United Kingdom

## Abstract

The amphibian decline and extinction crisis demands urgent action to prevent further large numbers of species extinctions. Lists of priority species for conservation, based on a combination of species’ threat status and unique contribution to phylogenetic diversity, are one tool for the direction and catalyzation of conservation action. We describe the construction of a near-complete species-level phylogeny of 5713 amphibian species, which we use to create a list of evolutionarily distinct and globally endangered species (EDGE list) for the entire class Amphibia. We present sensitivity analyses to test the robustness of our priority list to uncertainty in species’ phylogenetic position and threat status. We find that both sources of uncertainty have only minor impacts on our ‘top 100‘ list of priority species, indicating the robustness of the approach. By contrast, our analyses suggest that a large number of Data Deficient species are likely to be high priorities for conservation action from the perspective of their contribution to the evolutionary history.

## Introduction

The current biodiversity crisis demands pragmatic triage solutions. Lists of priority species are an important tool for the effective allocation of scarce conservation resources. Such lists are typically dominated, at the national and global scales, by species of high conservation concern, usually those in the Endangered and Critically Endangered categories of the IUCN Red List. Increasingly however, the notion that species’ contribution to phylogenetic diversity should also be considered, has been gaining traction [1]–[5].

Amphibians are in the grip of an unprecedented extinction crisis [6]. One third of species are listed as threatened and a quarter are categorised as Data Deficient. Around 43% of species are considered to be in decline [7]. Large scale declines have occurred over the last few decades [8], and future decades are expected to see the extinction of many hundreds of species [9], [10]. The amphibian extinction crisis has been attributed variously to habitat loss and fragmentation [11], disease [12], [13], environmental contamination [14], overexploitation [15], introduced species [16], climate change [17], [18], and interactions between multiple threats [19]–[24].

Faced with this crisis, a set of conservation priorities for amphibian species is urgently needed. At present, only the three IUCN categories of extinction risk can distinguish among the approximately 2000 threatened species, of which over 400 are Critically Endangered. In this paper, we generate a set of global priorities for amphibian conservation based both on threat status and phylogenetic position using the currently available data. We show that a working hypothesis for the species level phylogeny of the entire class of nearly 6000 species can be generated from a small number of synthetic sources, namely a cladogram of higher taxa and an authoritative taxonomy. We calculate species ‘evolutionary distinctiveness’ (ED) scores based on this phylogeny, and combine them with categories of extinction risk to generate an ‘EDGE’ list for all amphibians. We present sensitivity analyses to test the robustness of our priority list to uncertainty in both sources of data used to compile them: the branching structure of the phylogeny and the categorization of species’ extinction risk. We also explore the impact of different choices about the way in which EDGE scores are generated from the combination of phylogenetic and extinction risk assessment data.

## Materials and Methods

Our phylogeny is largely based on three sources: the amphibian ‘tree of life’ described by Frost et al. [25], the species-level taxonomy of Amphibian Species of the World (ASW) [26], and the molecular phylogeny of Roelants et al. [27]. Species’ extinction risk categories were extracted from the Global Amphibian Assessment (GAA) [6]. In cases where the species taxonomy of the GAA deviated from that of the ASW, we treated the ASW as authoritative.

Our general aim was to produce a phylogeny that was both maximally inclusive (i.e. containing nearly all amphibian species) and maximally resolved (given the available data). Achieving this goal necessitated a number of ad hoc decisions about the placement of certain species and the precise nature of the branching patterns, and for many clades the desire for inclusivity was in conflict with the desire for phylogenetic resolution. For this reason, we designed some of our analyses to address directly the issues around uncertainty in the phylogenetic position of large numbers of species.

### Higher-level Topology

The primary source of topological information was the amphibian ‘tree of life’ described by Frost et al. [25] in a large monograph. The phylogeny, depicted in their figure 50, is based on both morphological and molecular data: it contains 526 tips, most of which correspond to amphibian genera, and is almost fully resolved, containing 522 internal nodes.

We pruned the Frost et al.’s [25] ‘tree of life’, to produce a ‘higher taxon tree’ to which assignment of ASW species would be relatively uncontroversial. A total of 169 tips were pruned. This includes the speciose genus Litoria, of which the Frost et al. phylogeny includes just 10 out of 162 species: our higher taxon tree contains just a single tip for the entire genus. Likewise, about 1/3 of the 169 pruned tips were in the speciose families Ranidae and Bufonidae.

We then added a 23 additional clades that were not included in Frost et al. [25]. From the ‘Comments’ field in ASW we placed *Chiropterotriton*, *Crossodactyloides*, *Cynops*, *Frostius*, *Kurixalus*, *Leptobranchella*, *Salamandrina*, *Spelaeophryne*, *Zachaenus*, and the *Leptodactylus pentadactylus* and *Triturus vulgaris* groups. From Roelants et al. [27] we placed *Caudacaecilia*, *Glyphoglossus*, *Hylophorbus*, *Luetkenotyphlus*, *Microcaecilia*, *Praslinia*, *Proteus* and *Xenorhina*. Finally, we placed *Onychodactylus* and *Protohynobius* from Zhang et al. [28], *Itapotihyla*, *Megastomatohyla* and *Tepuihyla* from Faivovich et al. [29] and *Barygenys* from Van Bocxlaer [30].

### Species-level Topology

We assigned each species in ASW [26] to each one of these higher taxa. In most cases, this was straightforward because the tips of the higher taxon tree were mostly at genus level. Generally, we used a star phylogeny i.e. an unresolved multifurcating tree for species within higher taxa. For genera containing subgenera or ‘species group’ names in ASW, we treated these taxonomic units as monophyletic clades, thus providing extra resolution. However, this introduced problems for some large genera in which not all species have been assigned membership to any subgenus or species group. We decided assignment to a genus under ASW represented valid phylogenetic information, so we sought ways to include these ‘orphan species’ without losing the additional resolution provided by this additional information. Our approach depended on the size of the genus and the number of intra-genus clades. For the large genera *Philautus* (145 species) and *Platymantis* (55 species), both of which contain species groups that include around two thirds of their species complement, we assigned the remaining third to an ‘orphan’ clade within each genus. For 163 species in 18 genera where the proportion of orphans was relatively small, we assigned the orphans to species groups at random. This included members of *Eleutherodactylus* (n = 89 orphans out of 483 species), *Rhacophorus* (24/70) and *Xenopus* (7/16).

For some taxa, material in ASW indicated that phylogenetic data was available to add further resolution. In some cases this was a simple observation of relatedness, e.g. ‘probable sister species’; in other cases it referred to an external study on the phylogeny of the group in question. We used all such information where available, combined with species group assignments (described above). For example, we used Emerson et al.’s [31] phylogeny of *Limnonectes* to generate resolution within species groups, for a total of 17 subgeneric clades: 24 species were assigned to one of these clades with confidence, 16 species were assigned to a random clade within known species group, and 10 were assigned completely at random.

Just three out of 382 higher taxa represent taxonomic units above the genus. These were the clades defined by the following species in Frost et al. [25]: *Argenteohyla siemersi*, *Hamptophryne boliviana* and *Phyllomedusa vaillantii*. We used ASW to determine which genera were likely close relatives, often based on their status in previous taxonomic monographs. We then treated these genera as monophyletic within the suprageneric tip, and assigned species to them as described above.

A total of 5713 species were assigned to higher taxa, representing around 97% of valid extant amphibian species and only 153 species could not be assigned to any of the higher taxa.

### Dating the Phylogeny

The ages of deep nodes come from Roelants et al. [27] who presented a molecular phylogeny of 171 amphibian species. Specifically, we used the version of Roelants et al.’s tree that was constrained to be compatible with Frost et al’s [25] tree of life (figure 3 in Roelants et al. [27]). Node ages below Roelants et al. were derived by assuming a ‘pure-birth model’ of cladogenesis (following [32], [33]). The pure-birth model is a popular null model of evolutionary diversification (e.g. [34]–[36]) and is based on a Markov process. Specifically, it estimates the age of a node as T * ln(a)/ln(b), where T is the age of the parent node and a and b are the number of species descended from the focal node and the parent node, respectively [32]. The full composite phylogeny can be found as supporting information online (Phylogeny S1).

### Evolutionary Distinctiveness and EDGE Scores

We estimated species’ contribution to phylogenetic diversity using the ‘Evolutionary Distinctiveness’ (ED) algorithm described by Isaac et al. [37], with a modification to the way in which scores were corrected for polytomies (nodes with >2 descendents). Isaac et al. used a statistical fit to simulated data in order to correct the ED scores of branches descended from polytomies. This correction factor decreases to zero for nodes with large numbers (>20) of descendants, leading to an underestimate of the ED of many species in poorly-resolved areas of the phylogeny (i.e. most species in our amphibian phylogeny). Instead, we used a ‘pure birth model’ of cladogenesis to derive a correction factor based on the expected (i.e. mean) ED, given all the possible resolutions of the polytomy [38]. This empirical correction factor yields ED scores that are almost identical to those derived from a recently-developed Bayesian method for resolving polytomies in dated phylogenies [4], [39].

**Figure 1:**
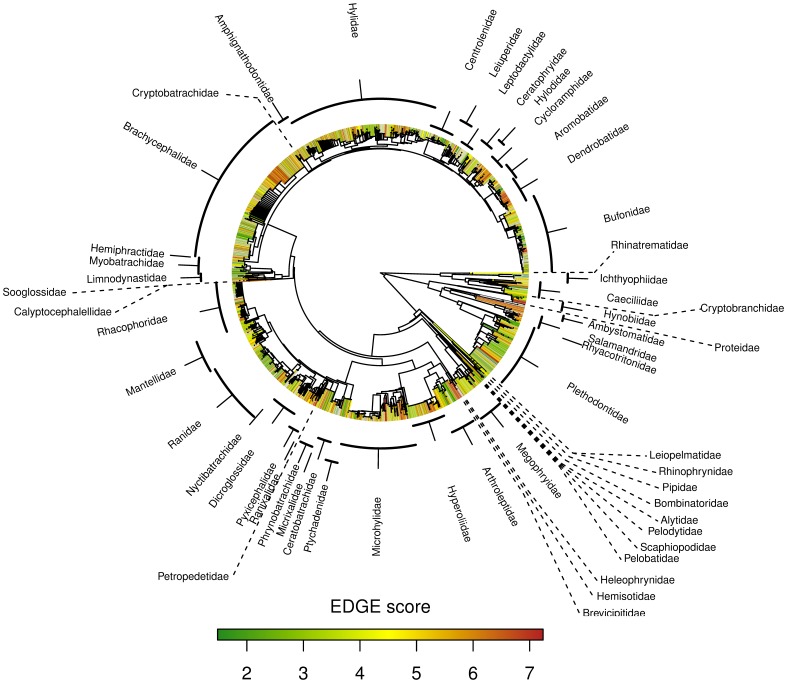
Species level phylogeny of 4339 amphibian species, colour-coded by species’ EDGE scores. Data Deficient and Extinct species have been omitted.

We calculated ED scores for each amphibian species using the caper package [40] in R [41]. Using the ‘EDGE’ algorithm previously used for mammals [4], [37], we combined these values with the extinction risk scores taken from Global Amphibian Assessment [6] to create our reference EDGE scores (figure 1). Data deficient species were excluded from this analysis. We created a further ‘candidate’ list of data deficient high ED scoring species (in the top 5% of ED scores) as targets for future threat assessment.

The ‘EDGE algorithm’ of Isaac et al. [37] is not the only way to combine ED scores with extinction risk categories, and the issue of how to convert these categories into an ordinal scale remains an issue [42], [43]. The EDGE algorithm treats each category as a quasi-probability in which each step is associated with increasing the extinction risk by a factor of two. The main alternative is the ‘expected loss’ (EL: [44]) algorithm, which is based on the actual probability of extinction over 100 years, using values of 0.1%, 1%, 10%, 67%, 99.9% for categories LC, NT, VU, EN and CR respectively, thereby giving much higher weight to CR and EN categories (compared with the EDGE approach). We compared the makeup of the top 100 species produced by both methods, and several variants thereof. One variant, named ‘IUCN500’ [42], is a modification of the EL approach but with extinction probabilities estimated over a much longer time period (i.e. 500 years), with probabilities of 0.5%, 5%, 39%, 99.6%, 100% [39]. The other two are variants on the EDGE calculation of Isaac et al. [37], in which extinction risk increases by 1.25 fold and 5 fold respectively, for each increase in threat categories. For each of these five methods, we expressed the makeup of the list as the running mean ED score of the top n ranked EDGE species, for all values of n from 1∶100. We compared these five empirical distributions two extreme selection criteria, one based solely on ED, the other selecting first all CR species then EN, in decreasing order of ED. Ideally, we would like a distribution that falls midway between these two extremes.

### Analyses and Simulations

We tested how uncertainty in the underlying data could affect the species chosen for conservation attention by the EDGE listing process. We examined the robustness of our priority list calculated using the standard EDGE algorithm, to four specific forms of uncertainty: a) the placement of species on the phylogeny (‘ED errors’), b) changes to species’ Red List status (‘GE errors’), c) future reassignment of species currently listed as Data Deficient (DD) and d) a sensitivity test varying the number of species for which there were errors in the data (i.e. 2% of the species have ED or GE errors compared to 25%).

We refer to the ED scores, extinction risk estimates and EDGE scores described above as the ‘unmodified’ or ‘reference’ sets. For each perturbation scenario (described in detail below), we generated 1000 replicate datasets at each level of perturbation and calculated EDGE scores for all species in each dataset. Given that the EDGE listing process has been previously been used to choose the top 100 ranked species to target for conservation attention [45], we used the similarity (i.e. the proportion of shared species) of the top 100 species as our overall measure of effect size.

#### a) ED errors: perturbing the phylogeny

The phylogeny is assembled in an ad hoc manner, so we wanted to be sure that our conclusions were robust to incorrect assignments. Assuming that most amphibian genera are monophyletic, the main errors in our phylogeny derive from treating subgeneric entities as clades, and the placement of species among these entities (see above). We simulated the impact of these decisions by altering the topology of the tree at random 1000 times. At each simulation, we selected 10% of species at random and moved them each to a different, but closely-related, clade. The severity of incorrect assignments was tested by sequential analyses moving another 1000 sets of randomly selected 10% of all species one, two, four, eight and finally sixteen clades away.

#### b) GE errors: altering the categories of extinction risk

We simulated the impact of uncertainty in each species’ extinction risk categorization. This is important because most changes on the Red List are due to advances in knowledge, rather than genuine changes in status [4]. For each simulation, we selected 10% at random and then moved them either up or down (again at random) one threat category (e.g. Vulnerable to Endangered or Near Threatened). This process was repeated allowing 10% of species to move two, three and then four categories up or down. In all cases, if a Least Concern status species was chosen to be moved down it was kept at Least Concern and, conversely, if a Critically Endangered species was chosen to be more severely threatened it was kept at Critically Endangered.

#### c) Data deficient species

Approximately a quarter of amphibian species are categorized as Data Deficient (DD) [6]. An unknown proportion of these species are, in reality, not at risk of extinction whilst others are likely to be threatened. To assess potential impact that DD species could have on EDGE scores, the DD species were randomly assigned threat categories at the same ratio of CR:EN:VU:NT:LC as for the set of species for which threat categories are known. We then repeated this simulation assuming that DD species were more threatened than expected. Again we randomly assigned threat categories at the same ratio as before, but then manually increased the newly-assigned threat categories by one level. We repeated the analysis three more times, first increasing each DD species newly-assigned threat level by two categories, and then also decreasing each by one and two levels respectively. Again, whenever Least Concern status was chosen to be less threatened, it was kept at Least Concern and, conversely, when critically endangered species were chosen to be more severely threatened; they were kept at that level. Unlike the other perturbations, in which species can either increase or decrease in EDGE score if selected, the simulated top 100 sets resulting from this process differ only in the number of currently DD species that displace the existing top 100.

#### d) Multiple sources of uncertainty

Finally, we tested how the total amount of uncertainty could affect the priority list. In the above scenarios, we changed 10% of species at random and examined each source of uncertainty separately: here we explore the effect of varying this number and include both perturbations to the phylogeny and changes to the extinction risk categories (i.e. both ED and GE errors), in order to test whether the uncertainty is additive or multiplicative. We simulated a scenario in which a proportion of species had been wrongly assigned by one or two threat categories and placed between one and two clades from their location on our reference phylogeny, with Data Deficient species treated as in c, above. We first chose 5% of all species randomly and altered their ED and/or GE scores as set out above. We repeated the analysis with the same parameter values but increased the number of species sequentially to 10, 15, 20, 30 and 40% of all species.

## Results

We calculated ED scores for 5713 amphibian species, of which 1344 were Data Deficient and 35 extinct, meaning that we could calculate EDGE scores for 4334 species (figure 1, for details see Table S1). The top scoring species was Archey’s Frog, *Leiopelma archeyi*, a Critically Endangered (CR) frog from New Zealand, followed by the Chinese Giant Salamander, *Andrias davidianus* (also CR, see supplemental material). The only non CR species in the top ten was the Purple Frog, *Nasikabatrachus sahyadrensis*, as it has the 7th highest ED score across all amphibians and is considered as Endangered (EN) by the IUCN. Of the top 100 species, 75 were classified as CR, 15 EN and 10 vulnerable (VU). There were 47 ‘candidate’ (DD but high ED) species, all but 10 of which are caecilians (table 1). The frog species *Hymenochirus boulengeri*, *Hymenochirus feae*, *Mixophyes hihihorlo* and the salamanders *Ambystoma flavipiperatum*, *Ambystoma rivulare*, *Ambystoma silvensis*, *Protohynobius puxiongensis* were the highest-ranking non-caecilian candidate species.

**Table 1:** The 47 candidate amphibian species with high ED scores and “Data Deficient” IUCN assessment staus.

*Rank*	*Family*	*Species*	*ED score*
1	Rhinatrematidae	*Epicrionops columbianus*	81.3908
2	Rhinatrematidae	*Epicrionops lativittatus*	81.3908
3	Rhinatrematidae	*Epicrionops marmoratus*	81.3908
4	Rhinatrematidae	*Epicrionops parkeri*	81.3908
5	Rhinatrematidae	*Epicrionops peruvianus*	81.3908
6	Caeciliidae	*Herpele multiplicata*	73.1665
7	Caeciliidae	*Luetkenotyphlus brasiliensis*	63.6999
8	Caeciliidae	*Geotrypetes angeli*	59.3842
9	Caeciliidae	*Geotrypetes pseudoangeli*	59.3842
10	Caeciliidae	*Boulengerula changamwensis*	56.8488
11	Caeciliidae	*Boulengerula denhardti*	56.8488
12	Caeciliidae	*Boulengerula fischeri*	56.8488
13	Pipidae	*Hymenochirus boulengeri*	52.5783
14	Pipidae	*Hymenochirus feae*	52.5783
15	Myobatrachidae	*Mixophyes.hihihorlo*	50.1187
16	Caeciliidae	*Dermophis costaricensis*	50.0494
17	Caeciliidae	*Dermophis glandulosus*	50.0494
18	Caeciliidae	*Dermophis gracilior*	50.0494
19	Caeciliidae	*Dermophis oaxacae*	50.0494
20	Caeciliidae	*Dermophis occidentalis*	50.0494
21	Caeciliidae	*Microcaecilia rabei*	49.7193
22	Caeciliidae	*Microcaecilia supernumeraria*	49.7193
23	Caeciliidae	*Gegeneophis carnosus*	45.7398
24	Caeciliidae	*Gegeneophis danieli*	45.7398
25	Caeciliidae	*Gegeneophis fulleri*	45.7398
26	Caeciliidae	*Gegeneophis krishni*	45.7398
27	Caeciliidae	*Gegeneophis seshachari*	45.7398
28	Caeciliidae	*Gegeneophis madhavaorum*	45.7398
29	Caeciliidae	*Gegeneophis nadkarnii*	45.7398
30	Ambystomatidae	*Ambystoma flavipiperatum*	42.3185
31	Ambystomatidae	*Ambystoma rivulare*	42.3185
32	Ambystomatidae	*Ambystoma silvensis*	42.3185
33	Hynobiidae	*Protohynobius puxiongensis*	42.1579
34	Caeciliidae	*Siphonops insulanus*	41.7074
35	Caeciliidae	*Siphonops leucoderus*	41.7074
36	Caeciliidae	*Crotaphatrema bornmuelleri*	37.1099
37	Caeciliidae	*Crotaphatrema lamottei*	37.1099
38	Caeciliidae	*Crotaphatrema tchabalmbaboensis*	37.1099
39	Caeciliidae	*Atretochoana eiselti*	35.9600
40	Ichthyophiidae	*Uraeotyphlus interruptus*	35.3800
41	Ichthyophiidae	*Uraeotyphlus malabaricus*	35.3800
42	Ichthyophiidae	*Uraeotyphlus menoni*	35.3800
43	Ichthyophiidae	*Uraeotyphlus narayani*	35.3800
44	Ichthyophiidae	*Uraeotyphlus oxyurus*	35.3800
45	Mantellidae	*Wakea madinika*	34.9872
46	Microhylidae	*Adelastes hylonomos*	30.5161
47	Limnodynastidae	*Notaden weigeli*	29.3150

**Figure 2:**
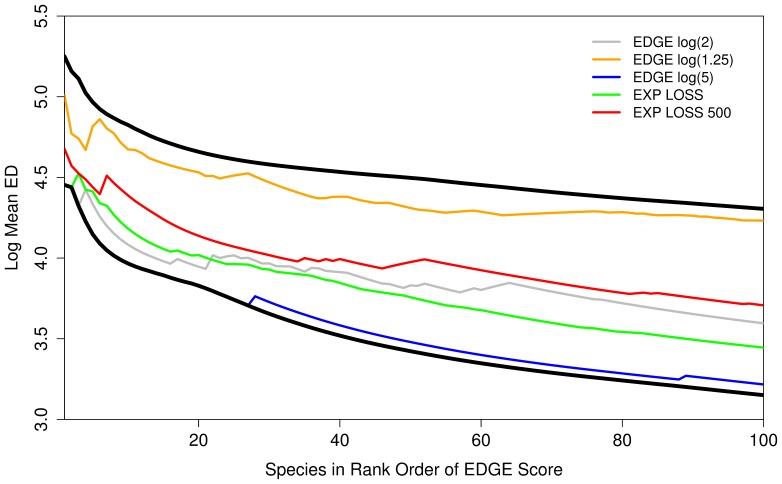
The mean ED scores of the top 100 species chosen using five different methods to create EDGE lists. Thick black lines indicate upper and low limits where species are chosen purely by having the highest ED score irrespective of threat (upper line) and just the most threatened (lower line) species are chosen. Lines represents the mean ED of the top 1:n top ranked species by each EDGE listing process. Note logarithmic y-axis.

Different listing procedures produced ranking lists with different weighting of the two component values of the EDGE listing approach (figure 2). The weighting used for the mammal EDGE prioritisation (‘EDGE log (2)’) in amphibians struck a reasonable balance between threat and ED for much of the top 100, but is slightly biased towards the threat component. Expected loss (EXP LOSS) showed a similar pattern of slight bias towards threat status as the EDGE list based on the log(2) listing. The approach that appears to take the most even-handed choice of species, with respect to the two input variables, is the Expect Loss approach used with probabilities that predicted 500 years into the future (Exp Loss 500).

**Figure 3:**
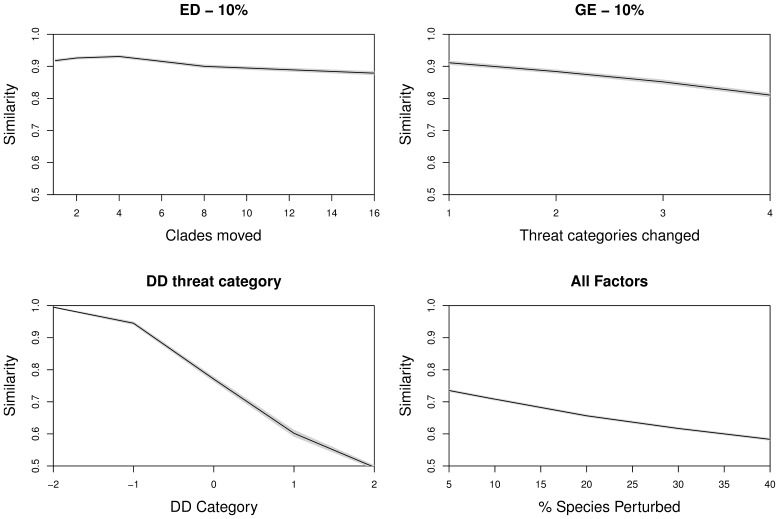
Results from simulations to explore the impact of uncertainty on the makeup of 100 highest ranked EDGE amphibian species. In each case, ‘similarity’ is the proportion of species shared with the unperturbed reference set, based on 1000 simulated datasets. Confidence intervals are drawn in grey but lie too close to the mean to be visible. Panel a) shows the impact of perturbing the evolutionary distinctiveness component (ED) by moving 500 (10%) randomly-selected species to closely related clades. Panel b) shows a similar relationship when 500 species have their threat categories perturbed. Panel c) shows the effect of different assumptions about true threat categories of Data Deficient (DD) species: with ‘DD category = 0′, DD species were assigned randomly, according to the distribution of non-DD species; with DD category >0 we assume that DD species are on average more threatened than expected. Panel d) shows the impact of multiple perturbations, with increasing the numbers of species perturbed. See text for further details.

Our perturbation of species’ ED and GE scores had very little impact on the makeup of the ‘EDGE top 100’ (figure 3). Small perturbations (2 clades or 2 threat categories) changed only a small proportion of the priority list (similarity = 0.9). Even under severe perturbation of 10% of the species' ED or GE values, the top 100 of the original EDGE list maintained a similarity of 0.85 with the reference set of unperturbed scores (figure 3 top left and right panels). The impact of Data Deficient (DD) species is much greater: when assuming that DD species were as threatened as expected (DD category = 0 on figure 3 lower left panel) then the similarity was 0.8 on average (in other words, 20 currently DD species would be listed in the top 100), but similarity dropped to 0.5 if DD species are on average two categories more threatened than expected.

When all three forms of uncertainty were combined, the similarity was lower still (figure 3 lower right panel). Low levels of both ED and GE errors (2 clades and 2 threat categories for 10% of species), plus assignment of DD species in the expected proportions, yielded similarity of around 0.7, which is roughly what would be expected from running each perturbation separately. Under the extreme scenario where 40% of species were perturbed, similarity dropped further, but only to around 0.6. In other words, quadrupling the level of perturbation causes just 10 changes to the makeup of the top 100 EDGE species.

## Discussion

In view of the unprecedented species decline, particularly among amphibians, immediate conservation action is necessary. However, the high number of threatened amphibian species will likely overwhelm global conservation efforts and resources, even if these efforts were to be intensified dramatically. Conservation action therefore must prioritise its actions and focus its attention and resources toward alleviating the situation for the most pressing cases. Basing prioritisation on phylogenetic uniqueness of species (ED), in addition to extinction risk status, captures not only the non-randomness of extinction (with respect to phylogenetic position), but also the fact that evolutionarily distinct species could have important ecological roles and that their loss would result in an over-proportional loss of evolutionary history [46], [47]. Here, we provide such a prioritization for the entire Class Amphibia. Our analyses show that the set of priority species is robust to the ad hoc nature of our phylogenetic tree and uncertainties in the extinction risk assessment of large numbers of species.

Our ‘EDGE list’ of amphibians is already a focus for conservation activities (http://www.edgeofexistence.org). This is important because threatened amphibians with high ED are no more likely to receive conservation mitigation than by chance, and just 15% of the top 100 high EDGE scoring amphibian species threatened with extinction are receiving active conservation attention [48]. The EDGE Amphibians project has supported conservation efforts and capacity building for over 15 top priority species (including *Andrias davidianus* in China, *Boulengerula niedeni* in Kenya, *Rhinoderma darwinii* in Chile, *Proteus anguinus* in Croatia and *Nasikabatrachus sahyadrensis* in India), funding training initiatives and conservation actions, with even greater aims to continue expanding the project’s scope of activities into the future. The EDGE Amphibians project has increased global awareness of amphibian species, providing international audiences with further reasons to become interested in lesser known species and amphibians in general. The project has thus far raised over £2 million for amphibian conservation initiatives around the world and the EDGE listing has played a major role in raising the profile of poorly known but highly distinctive species internationally. The EDGE website provides full details of high-priority species and ongoing conservation activities, and has proved to be a useful platform in leveraging support for amphibian conservation, illustrating how a science-based conservation prioritisation tool focusing on evolutionary distinctiveness can capture the interest of a wide range of conservation supporters and stakeholders. Whilst our focus here, and on the EDGE website, is on the highly-threatened species making up the top 100, the full has wider applications for conservation, such as mapping global hotspots of evolutionary distinctiveness and EDGE.

The production of our amphibian EDGE list was only possible by first assembling a species-level phylogeny. Whilst our approach is somewhat ad hoc, it is consistent with the principles of phylogenetic ‘supertree’ construction [49], [50]. Although in the future we can expect to obtain more accurate phylogenies based on molecular data, conservation must act in a timely manner given the urgency of the situation and the very real risk of imminent amphibian species extinctions globally. A complete molecular phylogeny of amphibians is unlikely to be available for many years, despite the enormous pace of developments in the molecular biology and bioinformatics, by which time it is likely that many species will have gone extinct [8], [9]. The phylogeny that we have produced will be a valuable tool for comparative studies of extinction risk [51], [52] and the randomness (or otherwise) of extinction risk [51], [53], as well as questions about the evolutionary history of amphibians [54]–[56]. Eventually, the combination of spatial, environmental and phylogenetic information could be used to predict the potential threat status of Data Deficient species [57].

Our simulations showed that even substantial amounts uncertainty about species’ phylogenetic position and threat status have only a minor on the set of priority species identified by the EDGE approach. Wholesale changes to the mammal taxonomy and reassessment of all species’ Red List status led to a change in the identity of around 15 species making up the top 100 EDGE mammals [4] (i.e. similarity = 0.85). Taxonomic and Red List instability are likely to be greater for amphibians than mammals, due to substantial uncertainty around cryptic species complexes in the tropics [54], [55]. Our perturbation of the input data has shown the top 100 species are rather resilient to errors and increased knowledge. The J-shaped distribution of ED scores is likely to be the main reason for this, as although the highest ED score is around 190 million years, only 5% of species have scores greater than 25 million years and 75% of species have scores under 12.5my. Therefore, if assessed and threatened, the small number of highly distinct species will remain in the top 100 unless a serious mistake has been made in the phylogenetic (and likely morphological) analyses of these species.

By far the most substantial source of uncertainty in our analyses surrounds the true conservation status of species currently listed as Data Deficient. Our list of ‘candidate’ species should be targeted for data collection in order to make full Red List assessments as a matter of urgency. The candidate list is dominated by caecilian species, which are typically cryptic and poorly understood. The whole group is in need of major taxonomic reassessment before detailed conservation targets can be established [25]. Reassuringly, their principally fossorial nature means that they may be, in many cases, relatively common but undetected [56]. If true, this would be a rare piece of good news among the devastation of amphibian biodiversity that continues all around us. In practical terms, the EDGE approach can successfully catalyze conservation action for little known and often overlooked amphibian species. It is proving itself to be a very useful prioritization tool in the development of conservation initiatives and also has considerable potential to continue raising awareness of the plight of amphibians globally.

## Supporting Information

Table S1 **EDGE and ED scores of all amphibians (see text for details).**
(CSV)

Phylogeny S1 **The composite phylogeny dervied as described in the text to build the EDGE and ED scores with.** The file can be read and converted in other formats using the open source programming environment R using the read.tree function of the library ape.
(TRE)
